# Piezo2-Initiated Ultrafast Signaling and Its Acquired Channelopathy in Light of Quantum Gravity Theory

**DOI:** 10.3390/ijms27094129

**Published:** 2026-05-05

**Authors:** Balázs Sonkodi

**Affiliations:** 1 Department of Health Sciences and Sport Medicine, Hungarian University of Sports Science, 1123 Budapest, Hungary; bsonkodi@gmail.com; 2 Department of Sports Medicine, Semmelweis University, 1122 Budapest, Hungary; 3 Faculty of Health Sciences, Institute of Physiotherapy and Sport Science, University of Pécs, 7624 Pécs, Hungary; 4 Physical Activity Research Group, Szentágothai Research Centre, 7624 Pécs, Hungary

**Keywords:** ultrafast signaling, gravity, mechanotransduction, proprioception, wormhole-like mechanism, acquired Piezo2 channelopathy, cryptochrome

## Abstract

Big Bang theories are connected to gravity by force of attraction. Forced lengthening, like eccentric contractions, instigate proprioception as a result of working against gravity. Piezo2, as the principal mechanosensory ion channel responsible for proprioception, is theorized to fine-modulate these anti-gravitational contractions in order to provide system-wide ultrafast postural control. This mechanism may instantaneously emit energy and force through Piezo2 in order to offset gravity by anti-gravity entropic-spring-like stochastic mechanics and it is suggested to be propagated by quantum tunneling of protons (and electrons). However, a Piezo2-initiated wormhole-like mechanism with the contribution of cryptochromes should be considered as part of this ultrafast long-distance non-synaptic neurotransmission, although the quantum gravity concept is short of being unequivocally proven to be unified with quantum theory. The impairment of this theoretical ultrafast signaling is analogous to a Big Bang-like mechanism within a given compartment, or acquired Piezo2 channelopathy, leading to the principal gateway to pathophysiology.

## 1. Introduction

Gravitational waves are induced at any oscillatory frequencies by the relative motion of gravitating masses with propagation at the speed of light [1]. Moreover, waves like sound waves and electromagnetic waves propagate energy, momentum and angular momentum away from its origin [1] and gravitational waves are not different in this aspect. The Nobel Prize was awarded to Rainer Weiss, Barry C. Barish and Kip S. Thorne in 2017 for detecting these gravitational waves. However, the quantum gravity concept is short of being unequivocally proven to unify with quantum theory. Furthermore, it is suggested that the force of gravity evolves as an entropic force and that this is the result of changes in the entropy stemming from the positions of material bodies in space [2].

This perspective manuscript is meant to introduce that a “Big Bang”-like mechanism-induced energy blast exists within the human body in the form of an acquired Piezo2 channelopathy and oxidative phosphorylation (OXPHOS) depletion. As an underlying theory, Piezo2 may function as an ultradian ultrafast sensor and fine modulator to counterbalance energy and force in order to offset gravity (Table 1). Accordingly, the current author proposes that forced lengthening, e.g., eccentric muscle contractions, could create such an instantaneous gravitational force offsetting counterbalance. Piezo2 ion channels may instigate ultrafast high yielding energy generation by OXPHOS-derived proton motive force and ATP in order to fine-modulate anti-gravitational force and energy, e.g., on large fiber oscillatory glutamatergic Ia type proprioceptive terminals. In support, it has been theorized that not only the force-from-lipid or force-from-filament principle may count in force-gated Piezo2 ion channel activation and modulation, but force-from-proton as well [3,4]. Noteworthy is that recent research suggests that Piezo2 might not be the main transducers of force in sensory neurons [5], but the author of the current paper proposes that Piezo2′s principality may exactly come from proprioception-related mechanotransduction by fine or ultrafast tuning of force and energy against gravity.

## 2. Piezo2 and Acquired Piezo2 Channelopathy as a Big Bang-like Mechanism

Mechanotransduction is the conversion of external physical cues to internal biological and chemical ones. The principal ion channel in mechanotransduction responsible for proprioception is claimed to be Piezo2 [6]. Note that some scientists question the principality of Piezo2 in proprioception and indeed some other ion channels contribute to it as well, like ASIC3 [7], Nav1.1 [8] and ASIC2 [9]. However, it is wort considering that the activation of these channels are in hierarchical order and Piezo2’s function is at the top of this hierarchy [10]. Accordingly, a recent theory associated with this principality that no other mechanosensory ion channel could initiate ultrafast proton-based long-distance synchronous signaling in the nervous system almost instantaneously, like Piezo2 [11]. Interestingly, the same paper that questions Piezo2 as the main transducer of force in sensory neurons shows that the presence of PIEZO2 is needed among mechanically gated channels for the very fast activation (and inactivation) kinetics of rapidly adapting mechanosensitive currents [5]. Proprioception, the timely conscious and unconscious positional sense of our extremities, had been a mystery as our “sixth sense” for almost 200 years, when Sir Charles Bell described it in 1830. Therefore, the Nobel Laurate Ardem Patapoutian and his team made a major contribution in bringing proprioception to light by identifying Piezo proteins, and especially Piezo2 [6].

Piezo2, and its related body-wide Piezo2 system, has been postulated to function like a protective “airbag” [4,11]. Correspondingly, once the ultrafast crash sensors of “airbags” face above-threshold rapid changes in speed and other stimuli, like compression/indentation and stretch, from collision then an inflator is ignited and inflates a bag in order to counter-cushion and protect the affected individual against gravity and direct injury. Indeed, the intrinsically disordered domain 2 (IDR2) of the Piezo2 protein structure is in control of velocity sensitivity of tactile stimuli, while IDR5 and IDR4 control membrane indentation and pressure-induced membrane stretch stimuli respectively [12]. However, the bag deflates immediately right after the crash and as a result the impact is absorbed. Let us consider that non-contact injuries, like the vast majority of anterior-cruciate ligament injuries (ACL) and delayed-onset muscle soreness (DOMS)—also suggested to be a non-contact injury [10]—entail a similar underlying primary damage mechanism, where the acquired microdamage of Piezo2 function may evolve [10,13]. As an analogy, strenuous or/and unaccustomed eccentric contractions under acute stress response (ASR)-induced prolonged over-excessive indentation/stretch (which is also compression on the Piezo2-containing annulospiral terminal of proprioceptive nerves in the muscle spindle) may posit the primary damage in the case of DOMS [4]. Excessive axial compression force under ASR in the case of non-contact ACL injury is theorized to be the initiating microdamage that ruptures the “airbag”, the equivalent of an acquired Piezo2 channelopathy [10], or a Big Bang-like mechanism. This “rupture” is proposed to be a “proton affinity switch” or “proton reversal” of Piezo2 in association with OXPHOS depletion [4,10,11]. As a consequence of the lost “airbag protection”, the selective barrier of the muscle spindle will be compromised due to the impairment of Piezo2–Piezo2 and Piezo2–Piezo1 crosstalk [10]. Consequently, the intact Piezo2-containing proprioceptive terminals of muscles spindles are the airbags of the extrafusal muscle space. However, this bi-compartmental mechanism not only exist in muscles, but also, for example, in the skin where the airbag is the Merkle-cell neurite complex—or entheseal compartments are the airbags for the spine, or enterochromaffin cells and their glutamatergic sensory innervation are the airbags for the gut [10]. Therefore, non-contact injuries cannot prevail in the presence of intact airbag or somatosensory/proprioceptive terminal Piezo2, and consequently acquired Piezo2 channelopathy presents the proposed preceding Big Bang-like mechanism.

Up to now three studies have provided direct evidence that acquired (not inherited) Piezo channelopathy exist, after it was first put forward by the current author in 2021 [14]. Two of these studies revealed the presence of acquired Piezo2 channelopathy in Angelman syndrome, a rare genetic disorder affecting the nervous system and proprioception, in which no inherited Piezo2 channelopathy has been detected [15,16]. The most recent one is an intriguing study that introduced inherited Piezo1 channelopathy with genetic manipulation that mimicked acquired Piezo1 channelopathy pathophysiology in Alzheimer’s disease (AD) and cerebral small vessel disease [17]. It is important to note that the primary damage in AD, and in other neurodegenerative diseases like amyotrophic lateral sclerosis (ALS), has previously been theorized by the current author to be irreversible acquired Piezo2 channelopathy on glutamatergic nerve terminals contributing to proprioception [3]. Hence, the mimicked acquired gain-of-function Piezo1 channelopathy in AD brain microcirculation [17] is suggested to be the direct consequence of the irreversible loss-of-function Piezo2 channelopathy due to impaired Piezo2–Piezo1 crosstalk [10]. This impairment could evolve bi-directionally, so from the direction of acquired Piezo2 or Piezo1 channelopathy as well, but Piezo2 represents the principal conductor of this Piezo crosstalk on glutamatergic nerve terminals contributing to proprioception [10]. Interestingly, systemic administration of PIP2, a phospholipid with high negative charge, rectified acquired Piezo1 channelopathy [17], as PIP2′s critical role in acquired Piezo channelopathy was highlighted earlier by the current author [10]. Moreover, supplementation of linoleic acid, a polyunsaturated omega-6 fatty acid with a negative charge, provided a remedy to acquired Piezo2 channelopathy in Angelman syndrome, too [15]. Correspondingly, it has been suggested earlier that the depletion of negatively charged lipids, like cholesterol and PIP2, under prolonged over-excitation under allostatic stress of Piezo ion channels promote the evolvement of a proton affinity switch or acquired Piezo channelopathy [10]. The significant functional relevance of charge alteration in the vicinity of Piezo2 ion channels on route to a proton affinity switch or Piezo2 channelopathy is supported by genetic analysis from an ALS cohort [18].

In summary, Piezo2 function is a precisely orchestrated clockwork mechanism, as recent research demonstrates [19], in association with its very fast activation and inactivation mechanics [5]. These ultrafast precise functional characteristics puts Piezo2 at the top of a hierarchy, therefore verifying its role as the principal proprioceptive ion channel in mechanotransduction. The impairment of these precise mechanics, represented in acquired Piezo2 channelopathy, or the primary damage, could evolve due to prolonged forced lengthening under allostatic stress, leading to impaired proprioception, impaired anti-gravity protection and OXPHOS depletion, coined as a Big Bang-like mechanism.

## 3. Wormhole-like Mechanism, Recoil Energy and Entropy

**Figure 1:**
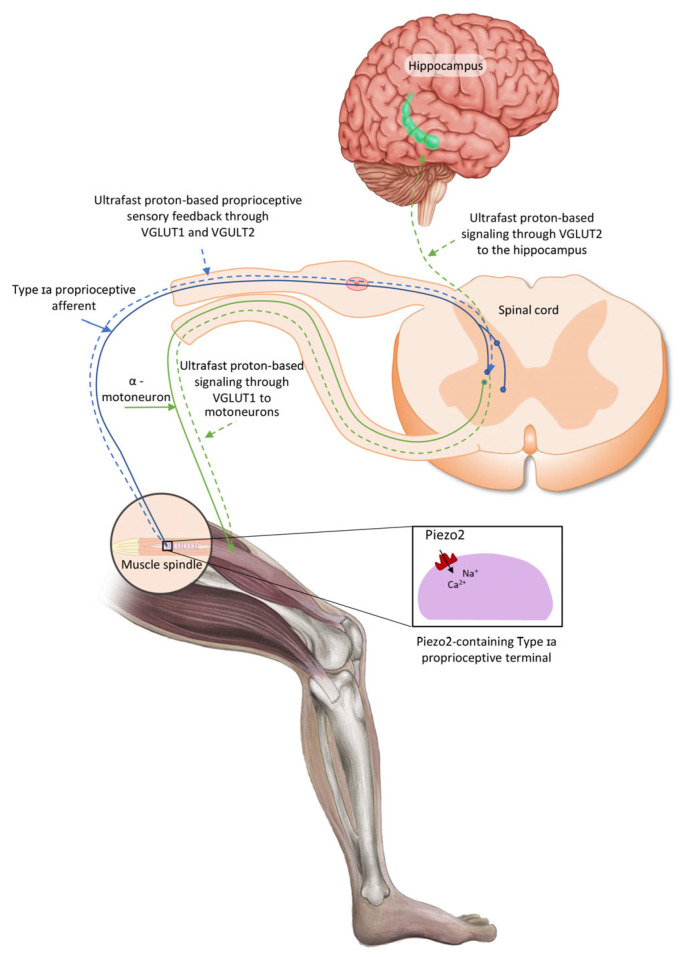
Conceptual proprioceptive pathways arising from intrafusal Type Ia proprioceptive afferent terminal Piezo2-initiated ultrafast proton-based long-range synchronization to hippocampal theta rhythm through VGLUT2 and to motoneurons through VGLUT1—the current figure is an English adaption of the figure from *Hungarian Rheumatology* [26].

The aforementioned oscillatory glutamatergic Type Ia proprioceptive terminals are located within the muscle spindles and surrounded by a fluid cavity with proposed functional relevance, and these muscle spindles are encapsulated by selective barriers [20]. It is important to consider that Piezo2 can sense pressure pulse transduction and this transduction could be set in motion through a closed rigid fluid-filled compartment, or chamber, almost right away at the speed of sound in agreement with Pascal’s law [21]. The Piezo2-containing muscle spindles, the gastrointestinal tract, the skin, the entheseal compartments of the spine, the cornea, the circulatory system, the atrium and ventricle of the heart and the brain, amongst others, are such compartments or chambers as well [10]. Therefore, compartmentalization with selective barriers is important; however, these underlying functional structures of the nervous system are largely unaccounted. Moreover, these compartments are hypothesized to be in functional cross-communication and principally cross-frequency, coupled by the Piezo system that entails the Piezo2–Piezo2, Piezo2–Piezo1 and Piezo1–Piezo1 cross-talks [10]. Proprioceptive pseudounipolar Ia sensory afferents evidently connect the muscle spindle to the spinal cord with the closed-gate intact blood–spinal cord barrier (BSCB) function under homeostasis [20]. Moreover, the fluid-filled cavity containing a muscle spindle under stretch is an analogous closed rigid fluid-filled compartment, as mentioned above, like the spinal cord, and it is functionally connected to the brain in a similar fashion. Accordingly, the pressure pulse detection is propagated by the force- and indentation/stretch-gated Piezo2 content of the peripheral terminals of intrafusal Ia proprioceptive afferents [22] at the speed of sound [21]. As a result, Piezo2-induced principal proprioceptive signaling is suggested to be transduced in a novel ultrafast and long-range fashion. This hypothetical proprioceptive signaling may be transduced by oscillatory glutamatergic Ia afferents toward the Piezo2-containing hippocampus via quantum tunneling of protons with the involvement of VGLUT2 and toward motoneurons through VGLUT1 [4,10,23]. In support, recent research showed that even motoneurons contain Piezo2 [24]. This ultrafast Piezo2-initiated non-synaptic long-distance neurotransmission toward the hippocampus is suggested to evolve along ultradian events as the ultrafast backbone of brain axes, like the eye–brain and auditory/vestibular–brain axes, beyond the aforementioned proprioceptive muscle–brain axis (Figure 1) [3,25].

It is important to note the distinct feature of eccentric or lengthening contractions that come with higher cortical excitation and lower motor unit discharge [27,28]. In addition, eccentric contractions absorb energy from an external load [29], support the body against gravity, absorb shock, and store recoil energy from ground reaction force (GRF) for accelerating contractions [27,30]. They have also been depicted as negative muscular work [29]. However, a problem arises when the storing of energy from the external load, coming from the eccentric contraction-based accelerating movement, cannot “recoil” in the decelerating movement due to the aforementioned “ruptured airbag” or acquired intrafusal Piezo2 channelopathy and resultant selective barrier disruption [31]. Consequently, the excess “unrecoiled” energy coming from accelerating eccentric movements may be partially absorbed by muscles and other tissues, like connective tissue, fascia and extracellular matrix, in a damaging way as part of the secondary damage [31]. In support, it has been shown that damaging eccentric exercise is to blame for the impairment of proprioception [32]. Indeed, one cardinal symptom of DOMS is impaired proprioceptive function right after eccentric exercise, proposed to arise from the muscle spindle [33]. Therefore, the aforementioned antigravitational ultrafast fine-tuning feature in association with eccentric contractions may principally arise from Piezo2-containing proprioceptive terminal loading with the involvement of the stretch reflex [34].

After all, it is worthy of consideration that the ultrafast Piezo2-initiated non-synaptic long-distance somatosensory neurotransmission toward the hippocampus along ultradian events may resemble an Einstein–Rosen bridge or a wormhole. A wormhole is a link between entanglement and gravity, as theorized by quantum gravity theory. Accordingly, these wormholes may connect two distant points through a tunnel in spacetime, meaning travel in space and time, and collapse almost instantly in the absence of negative energy [35]. Noteworthy is that there is no unequivocal evidence that wormholes exist [35] and certainly no proof of a link to quantum theory. However, these unstable tunnels may transduce information in a coordinate system. It is important to note that the clock of the two ends of such a wormhole may always stay synchronized regardless of how the ends move in space; hence, the constant time allows space-like separation on a surface. Notable is that previously it has been proposed that Huygens synchronization contributed to the synchronized state along the novel ultrafast Piezo2-initiated non-synaptic long-distance proton-based somatosensory neurotransmission toward the hippocampus [23], based on the work of Kocsis et al. [36]. Moreover, wormhole frequency coupling also exists in theoretical physics where holographic duality may arise from the coupling between two quantum systems [37]. This is also in line with the earlier theory that proton–proton frequency coupling through VGLUT2 may provide the ultrafast Piezo2-initiated non-synaptic long-distance somatosensory neurotransmission toward the hippocampus [23]. In support, conditional knockout VGLUT2 mice exhibited remarkably different oscillatory activity in the hippocampus with impaired spatial memory [38]. Above all, this hypothesized Piezo2-initiated wormhole-like mechanism not only could explain hippocampal spatial memory, but muscle memory as well, due to holographic duality. Furthermore, these Piezo2-initiated wormhole-like mechanisms may present the ultradian backbone of brain axes, not to mention the suggested ultradian clock of the hippocampus [3,25]. Moreover, a Piezo2-initiated wormhole-like mechanism may provide peripheral spatial and speed inputs to the space and speed encoding of the hippocampal theta rhythm, supporting locomotion, learning and memory, as was theorized earlier [23].

PIEZO2 is critical in the defensive arousal response (DAR) as recent traumatic brain injury (TBI) research showed [39]. It is important to note that the whiplash nature of mild TBI is suggested to have an analogous bi-phasic non-contact injury mechanism on the periphery, like DOMS and non-contact ACL injury, where the primary damage may arise from acquired proprioceptive afferent terminal Piezo2 channelopathy of neck muscle spindles [10]. DAR is essential for survival, and it is turned on by a perceived threat and evoked by visual and auditory cues in the presence of motor abilities [39]. DAR may be analogous to ASR and that is part of the neurocentric acquired Piezo2 channelopathy theory of DOMS and non-contact ACL injury [10,13] and might be induced often, e.g., during competitive game situations. Interestingly, a recent preprint paper theorizes the proton-based ultrafast matching/synchronization of the Piezo2-initiated eye–brain, auditory/vestibular–brain, and proprioceptive muscle–brain axes within the hippocampal hub [40], in line with the abovementioned PIEZO2-related DAR mechanism. Indeed, earlier research showed that sensory input could be temporally organized by ultradian brain rhythms in concert with temporary synchronization of the heart rate, medulla firing and the hippocampal theta rhythm [41,42]. Accordingly, the PIEZO2-related DAR study may highlight the ultrafast ultradian sensory and ultradian rhythm generation function of Piezo2 [43] in order to support postural stability instantaneously against gravity under DAR/ASR. This may explain why DOMS alters the response to postural perturbations [44] and significantly increases the medium latency response (MLR) of the stretch reflex [45], as a result of the suggested acquired Piezo2 channelopathy. Indeed, the aforementioned research paper revealed that in the absence of PIEZO2 the very fast neuronal current activation among mechanically gated channels was reduced [5], in support of the theorized ultradian ultrafast sensory function of Piezo2 [10]. Therefore, the hypothesized Piezo2-initiated wormhole-like mechanism under DAR/ASR may not only be analogous to the theorized underlying Piezo2-initiated proton-based ultrafast ultradian hippocampal backbone of eye–brain, auditory/vestibular–brain, and proprioceptive muscle–brain axes, but it may temporarily synchronize to the hippocampal hub and theta rhythm as well. Nevertheless, acquired Piezo2 channelopathy may impair this fine tuning of Piezo2-initiated ultrafast ultradian hippocampal synchronization, leading to reduced ability to respond to ultradian events, especially perturbations, therefore increasing injury risk, as is the case in DOMS for example [10].

Piezo2 has been called the principal cross-frequency-coupler, or entrainer, under allostatic stress [43]. Accordingly, an additional ultradian brain axis contribution should be considered within ultradian rhythms and that is the ultradian heart–brain axis. These ultradian heart–brain axes may fine-control the autonomic nervous system (ANS) regulation through Piezo2–Piezo2 crosstalk in a heart rate dependent manner [43], fully in line with the earlier observation of Pedemonte et al. [41,42]. It is important to note that Piezo2 channels have been associated with low-frequency Schottky semiconductor barrier diode-like function [23], as well as with a super-Schottky diode one [43]. Paired-associative electromagnetic stimulation, including both transcranial and peripheral, after a DOMS-inducing exercise had a positive therapeutic impact not only on DOMS-related symptoms [46], but also on heart rate variability (HRV) parameters [47], reflecting the ANS’s involvement in this proprioceptive impairment. This is in contrast to only peripheral electromagnetic stimulation when the treatment proved to be ineffective in DOMS [48]. The supportive finding of the Piezo2 channelopathy-involved neurocentric DOMS theory [10]—and the abovementioned therapeutic effect of paired-associative electromagnetic stimulation [47,49] that Piezo2 is found to be the underlying precise/fine mediator of magnetic stimulation [50]—was suggested earlier [10]. Presently it is an unattainable challenge to create a man-made gravitational wormhole due to the large negative gravitational energy demand. Nonetheless, magnetic materials with superior magnetic permeability, including superconductors, led to the remarkable scientific accomplishment that a magnetic wormhole was constructed in laboratory settings [51]. Noteworthy is that a magnetic wormhole is not identical to the Einstein–Rosen bridge (gravitational wormhole); however, it also connects two distant points to allow electromagnetic wave propagation via a magnetically undetectable and invisible tunnel, hence underscoring the mechanistic feasibility of the proposed ultradian ultrafast Piezo2-initiated non-synaptic long-distance neurotransmission.

The question rightly arises where the negative energy comes from in order to induce the proposed wormhole-like mechanism. In order to understand, it is worthy to consider that a hippocampal theta wave activates prior to moderate exercise onset [52]. Hence, there is a preliminary theta phase that at first hand could not be fully explained by proprioceptive Piezo2 activation. Indeed, two Piezo2-initiated ultradian backbone brain axes should be differentiated from this aspect, namely the eye–brain and the auditory/vestibular–brain axis. Accordingly, the speed of sound-like mechanotransduction could be easily interpreted along the Piezo2-initiated ultradian backbone auditory/vestibular–brain axis. But even more importantly, the Piezo2-initiated ultradian backbone eye–brain axis may allow an even faster wormhole-like mechanotransduction and could explain the aforementioned preliminary phase of hippocampal theta activation prior to moderate exercise onset. Correspondingly, not only Piezo2, but the co-functioning of cryptochromes should be taken into consideration in retinal ganglion cells (RGCs), as they are also present in photoreceptors [53]. The evolutionarily conserved blue-light photoreceptor cryptochromes, CRY1 and CRY2 in mammals, are critical to the circadian clock [54], but also interact with the ultradian rhythm and hippocampal function [55]. Interestingly, interleukin-6 deletion alters the number of ultradian activity bouts and in parallel alters CRY1 expression in the hippocampus as well [56]. Noteworthy is that earlier it was theorized that interleukin-6 increase is the direct consequence of acquired Piezo2 channelopathy [57] and the Piezo2-initiated ultrafast regulation of ultradian rhythm is also suggested in the hippocampus along the theorized hippocampal ultradian clock [3]. Moreover, cryptochromes contribute to hippocampal memory formation as a clock gene by regulating rhythmic transcription of plasticity-related genes [58]. Most importantly, cryptochromes, not only in birds [59] but in humans as well, serve the light-dependent magnetosensitivity [60]. The magnetoreception of birds serves as an inclination compass (“senses the axial course of the field lines and interprets their inclination in space”), it is narrowly tuned (“magnetic fields with markedly lower or higher (!) intensity cause disorientation”) and it requires short-wavelength light (“orientation is possible under light from ultraviolet to about 565 nm green”) [59]. Accordingly, a radical pair model was put forward [61] where in the case of absorbed photons either singlets with antiparallel spin or triplets with paralleled spin are formed [59]. The singlet/triplet ratio in relation to the magnetic field explains the inclination compass of birds [59]. The blue-light photoreceptor cryptochrome has been suggested to be this photon-absorbing magnetoreceptor [62,63].

However, the current author proposes that protons should be considered as ultrafast non-synaptic neurotransmitters of the mechanotransduction domain for the interplay between magnetoreception and proprioception, as was hypothesized earlier [10]. Indeed, a recent paper also described that magnetoreception, orientation and navigation (also part of proprioception) in the geomagnetic field are dependent on an ion forced oscillation mechanism by membrane voltage-gated ion channels [64]. As was put forward earlier, the voltage-gated non-selective cation channel Piezo2-containing oscillatory glutamatergic proprioceptive terminals are the principal forced peripheral oscillators that synchronize spatial, time and speed information to the hippocampal oscillator in an ultrafast fashion in support of proprioception, and hippocampal learning and memory [10,23,65]. Therefore, the current author proposes that the ultrafast cross-talk affected by radical pairs between RGC cryptochromes and Piezo2 may construct the suggested wormhole-like mechanism towards the hippocampal cross-talk between cryptochromes and Piezo2, and prefrontal cross-talk between cryptochromes and Piezo2. These ultrafast wormhole-like pathways may explain the abovementioned preparatory theta phase, reflecting the increased cognitive and motor alertness. In support, an interesting study implies the long-distance PIEZO-initiated mechanotransduction shows that PIEZO is indeed the magnetic field and blue-light sensor, as was theorized earlier by the current author [10,25], in order to promote root growth with the essential contribution of CRY1 and CRY2 [66]. Therefore, it seems that the cross-talk between cryptochromes and Piezo, Piezo2 in humans, is evolutionarily conserved and supports long-distance mechanotransduction.

It was suggested in DOMS that proton motive force is generated from proton-coupled electron transfer (PCET) [4] coupled to Grotthuss-type translocation of protons from interfacial water by Piezo2 [43]. Therefore, Piezo2 seems to be the coupler in this transfer of protons at a nanoscale distance that may span proprioceptive membrane terminals, as was proposed in reference to biological membranes without Piezo2 contribution [67]. Moreover, it has also been theorized that Piezo2 may contribute to the quantum tunneling of protons at long-distance through vesicular glutamate transporter 1 (VGLUT1) to motoneurons and to the hippocampus through VGLUT2 [4,23]. However, the negative energy of photons may come from negative absorption or negative frequency in line with quantum gravity theory, but this theoretical modeling is not the subject of this paper, which is rather to highlight the concerted cryptochrome and Piezo2-initiated ultrafast bi-directional pathways to the hippocampus (and to the prefrontal cortex) from RGC. Accordingly, the current author suggests that absorbed photon-induced radical pairs by cryptochromes are coupled to the Piezo2-induced quantum tunneling of protons, not to mention they may induce a quantum entanglement at long-distance in a wormhole-like fashion. Hence, this hypothetical mechanism at the speed of light may explain the preparatory theta phase prior to moderate exercise onset.

Forced lengthening contractions are also coined as negative work mainly stemming from GRF. However, it should be considered that the coupled absorbed photon-induced radical pairs by cryptochromes and the Piezo2-induced quantum tunneling of protons may also be part of the proprioceptive control of eccentric contractions. In order to understand the metabolic and energy generation mechanism of eccentric contractions, it is worth to consider that Piezo2 may modulate the reactive oxygen species (ROS)-dependent mitochondrial high frequency oscillations, and Piezo2 channelopathy might fail to do so [43]. In support, DOMS increases ROS production [68] and recent Piezo1-related research also show there is a link between Piezo ion channels and oxidative modulation [69]. However, the current author proposes that Piezo exerts fine oxidative modulation and not the other way around. Furthermore, ROS have a dual role both in hippocampal learning and memory, and in hippocampal neurotoxicity and even neurodegeneration [70,71]. This dual role of ROS is also present in the mitochondria of the heart as well [72], and may not only be telling about the underlying Piezo2-initiated wormhole-like mechanism (ultradian backbone) of the heart–brain axis, but about the Piezo2 crosstalk coupled to the ANS [25]. Acquired Piezo2 channelopathy theory posits that the primary damage may evolve at nerve terminals, like the Type Ia proprioceptive one (hence in RGC as well), where the mitochondria content is high, reflecting the high energy demand [20]. In addition, on route to this hypothetical microdamage is the critical pathway of electron leakage serving ROS production primarily through the electron transport chain [20,23] and proton motive force [4]. Acquired Piezo2 channelopathy theory proposes that it may come with a proton affinity switch [4]; hence, it might explain the dual role of ROS [43]. Acquired Piezo2 channelopathy was even hypothesized to be the principal gateway to pathophysiology, or the suspected primary damage, and that is the one common root cause of aging initiation [10]. In accordance with the dual role of ROS, increased ROS production may explain the inducement of the inflammatory reflex within homeostasis in support of remodeling, in contrast to the proton affinity switch that may induce the gateway reflex as a breach of remodeling [10]. Accordingly, Piezo2 channelopathy may not only instigate a proton affinity switch, leading to a neural switch or miswiring, but may also impair quantum tunneling of protons and electrons from mitochondria [4], leading to increased ROS production, and resultant increased entropy and accelerated aging on the chronic path [43]. Correspondingly, this pathophysiology is analogous to the one observed in aging of brain mitochondria [73]. In addition, non-linear parameters of HRV, such as entropy, also reflect upon the age dependence of HRV of the heart, likely due to age-related degradation of Piezo2 [74]. Finally, it is also worth considering that a proton affinity switch might explain why reverse electron transport could prevail [43]. Indeed, mitochondrial ROS production is increased by reverse electron transport [75]. Piezo1 is essentially involved in force-induced ATP secretion [76], as predicted in the case of Piezo2 as well [3]. Moreover, this force-induced ATP release may be coupled to proton motive force generation, while the theorized proton affinity switch or acquired Piezo2 channelopathy may result in transient OXPHOS depletion and the loss of proton motive force generation [4].

It bears further consideration that the low-frequency (LF) power of HRV has been proposed to reflect the Piezo2 activity level of baroreceptors with some residual Piezo1 activity contribution [34]. Part of this theory originated from the realization that LF power and entropy are counter-modulated where gravity has an implicated role [34]; however, DOMS (due to acquired acute transient Piezo2 channelopathy) and the theoretical longitudinal degradation of Piezo2′s principal function (due to acquired chronic Piezo2 channelopathy) impair this counter-modulation [43,74]. Moreover, this spring-like counter-modulation of LF power and entropy by Piezo2 has also been suggested [34] based on an earlier work showing that muscle spindles predominantly act like springs [77]. In contrast, other muscle spindle types function like breaks [77] that are suggested to be rather GABAergic by the current author. Indeed, a study later substantiated these Piezo functions by establishing their underlying entropic-spring-like mechanics that are tunable by frequency [78]. Moreover, this study also hypothesizes that other ligand-gated channels may exert spring-like mechanics in support of stochastic gating as well, such as acid-sensitive ion channels, N-methyl-d-aspartate receptors and others [78]. However, the current author is in agreement with the earlier theory that Piezo2 is the principal signaler at the top of a hierarchy, or the master modulator, of concerted entropic-spring-like mechanics that initiate stochastic gating [34,43,74]. In addition, the impairment of this stochastic gating due to acquired acute transient Piezo2 channelopathy theorizes why DOMS may alter the response to postural perturbations [44] and significantly increases the MLR of the stretch reflex [45], not to mention the increased injury risk of DOMS [74]. It is important to note again that acquired Piezo2 channelopathy could be translated as a proton reversal or proton affinity switch [4]. In support, direct and inverse piezoelectric effects exist that could be influenced by external factors, like induced electric currents and electromagnetic fields [79].

After all, the current author proposes, based on his earlier works, that Piezo2′s entropic-spring-like stochastic gating feature principally serves the ultrafast initiation of anti-gravity protection along a wormhole-like mechanism during ultradian events. In contrast, the acquired channelopathy of Piezo2 impairs this principal stochastic gating in association with OXPHOS depletion, leading to impaired or lost inducement of the proposed wormhole-like mechanism and lost anti-gravity protection (Big Bang-like mechanism) due to proton reversal or an inverse piezoelectric effect on Piezo2 function. Moreover, Piezo2 is theorized to be the principal/master conductor of this precisely concerted ultradian ultrafast anti-gravity protective mechanism not only on the cellular level, but also on inter-cellular [10,80], compartmental [10] and system-wide levels [10] as well. It is important to note that this theorized mechanism certainly involves other ion (ligand-gated) channels’ contribution downstream in a hierarchical order in support of proprioception.

## 4. Symmetry-Breaking, Non-Linearity and Stress

It has been proposed that the proton-release capability of Piezo2 is symmetry-breaking, leading to the collapse of the disordered symmetric state in order to accomplish an ordered but not symmetric state as excitation increases along acute intensive exercise loading [43]. The current author posits that this distinctive feature of Piezo2 may allow it to respond to ultradian events in an ultrafast fashion under DAR/ASR. The quantum mechanical outcome of symmetry-breaking in reference to the hypothesized wormhole-like axes is that it transforms from a harmonic oscillator wavefunction to a spectrum of quantum state [81]. This transformation may be important to prioritize and differentiate between brain axes in favor of the stress-induced higher loaded/excited ones, e.g., prioritizing the intrafusal proprioceptive one over the aforementioned other ultradian sensory wormhole-like brain axes that are temporally organized by ultradian brain rhythms in coordination with temporary synchronization of the heart rate, medulla firing and the hippocampal theta rhythm. This transformation of the wormhole-like mechanism to quantum state may allow the enhancement of hippocampal spatial encoding and memory in a differentiated way under DAR/ASR in order to enhance the precision of ultrafast memory and spatial representation in support of proprioception under a DAR/ASR [82].

The metabolic footprint of this space-time-dependent inhomogeneous perturbation of biochemical instability is diffusion-induced symmetry-breaking [83]. Accordingly, glycolysis may be considered as a spatio-temporal dissipative structure based on diffusion, leading to the form of sustained oscillations [83]. The current author suggests that the symmetry-breaking of the wormhole-like mechanism that transforms from a harmonic oscillator wavefunction to a spectrum of quantum state, may also induce a symmetry-breaking in the wavelike pattern of glycolysis by upregulating OXPHOS. However, a proton affinity switch or acquired Piezo2 channelopathy may switch to a state when glycolysis is preferentially used over OXPHOS due to OXPHOS depletion [4]. More precisely the resultant impaired intracellular proton gradient may switch mitochondrial energy metabolism from the evolutionarily superior energy-generating OXPHOS and glutamine respiration pathways to the mitochondrial glucose and glutamine fermentation pathways [4]. During fast growth, this glucose and glutamine respiro-fermentation could run parallel, like in cancer [84] and immune cells [57]. It is important to highlight again the relevance of negatively charged lipid depletion, like cholesterol and PIP2, in the acquired Piezo2 channelopathy mechanism since it essentially contributes to the complexity of the metabolic switch [10]. The author of this paper suggests that the resultant derailment of ATP and ADP concentration may cause further stress on the affected cells and associated mitochondrial destabilization that conserves the symmetry-breaking and the non-equilibrium phase transition until Piezo2 channelopathy is sustained. This is in line with the explanation of the Sel’kov model of glycolysis as a spatial dissipative symmetry-breaking instability structure [83].

However, the current author also proposes that it is important to distinguish four hierarchical phases of energy generation under allostatic stress. One phase is when OXPHOS and glutamine respiration pathways are coupled, or the equivalent of the harmonic oscillator wavefunction of the wormhole-like mechanism. The second phase is when OXPHOS and glutamine respiration pathways are de-coupled. This decoupling is the equivalent of transformation of the wormhole-like mechanism from a harmonic oscillator wavefunction to a spectrum of quantum state and this symmetry-breaking in the wavelike pattern upregulates OXPHOS. In this second phase, recoil energy may not be stored efficiently anymore in the form of ATP and proton motive force; hence, prolonged eccentric contractions may become damaging. The third phase is when Piezo2 is inactivated, but symmetry-breaking of the quantum state of the wormhole-like mechanism is sustained due to the switch to secondary proprioception, namely to type II intrafusal fibers with ASIC3 content, with underlying heavier metabolic duress and the instability of the wormhole-like mechanism. The current author also proposes that this might be the moment when intensive physical exercise shifts the brain away from theta rhythm towards gamma and beta rhythm. The fourth stage is the suggested Big Bang-like mechanism, or acquired Piezo2 channelopathy when the proton affinity switch not only depletes OXPHOS, but shifts glycolysis and glutamine respiration pathways towards the mitochondrial damaging glucose and glutamine fermentation pathways [4]. This fourth stage may not only cause the collapse of the wormhole-like mechanism, but cannot be induced again until Piezo2 channelopathy is present; therefore the fine (ultrafast) control of anti-gravity protection may be impaired for the time being. This is why DOMS is not only associated with skeletal mitochondrial damage [85] due to the metabolism and energy generation switch [4], but alters the response to postural perturbations [44] and mimics a positive Romberg-test due to impaired fine (ultrafast) control of proprioception [40]. Moreover, damaging eccentric contractions and DOMS increases insulin resistance [86], impairs orthostasis in a diabetes-like manner [87], and alters HRV due to impaired fine (ultrafast) control of insulin sensitivity [10] and the ANS respectively [43].

Noteworthy is that ultradian oscillations in the plasma level of glucose and insulin are evident in non-diabetic individuals [88], while these oscillations are decreased and less controlled in diabetic patients [89,90]. One interesting study applied a mathematical non-linear two-delay model on the glucose regulation mechanism and showed that stochastic effects may also contribute to this regulative mechanism [91]. In support, DOMS-related studies not only found impaired orthostasis in a diabetes-like fashion [87], but also detected non-linear alteration in HRV as well [43], not to mention the entropic spring-like stochastic gating of Piezo2 [78].

Nerve growth factor (NGF) is also subject to ultradian oscillations in the plasma levels of healthy individuals [92]. The scientific debate of what comes first exists in science in regard to the primary damage of DOMS, where one side demonstrated that increased NGF initiates the pathophysiology onset [93]. The other side of the debate however theorizes that acquired Piezo2 channelopathy comes first followed by increased NGF production by mesenchymal cells [4]. This elevated NGF production could be the result of sensory terminal Piezo2 channelopathy-derived switched/miswired signaling and impaired cross-frequency coupling of Piezo2–Piezo2 and Piezo2–Piezo1, leading to impaired Piezo1-driven cell orientation and adjustment [4]. Notable is that NGF is essential for neural survival, growth and maintenance, while it shows decline with aging [94].

János (Hans) Selye coined good stress as eustress and bad stress as distress [95]. Accordingly, two states of Piezo2 may persist under allostatic stress. Inactivated intact Piezo2 under allostatic stress represent “coupled” (intact Piezo2 crosstalk) good stress, while the acquired microdamage of Piezo2 may represent “decoupled” (impaired Piezo crosstalk despite modulation is taken over by secondary proprioceptive ASIC3 as an adaptive mechanism) bad stress [43]. In support, evolutionarily conserved Piezo buffers mechanical stress through modulation of intracellular calcium handling in the Drosophila heart and the functional mutation of PIEZO fails this mechanical stress buffering, leading to pathological remodeling [96]. After all, the impaired low-frequency Schottky semiconductor barrier diode-like feature of Piezo2 likely fails to modulate ROS-induced high-frequency oscillations, but even more importantly may fail to initiate quantum tunneling of protons (and electrons) and might fail to induce the suggested wormhole-like mechanism as a result of Piezo2 channelopathy or a Big Bang-like mechanism.

Briefly put, energy-consuming symmetry-breaking modulated by the theorized ultrafast precise anti-gravity entropic-spring-like stochastic mechanics of Piezo2 provides non-linear system protection against gravity under allostatic stress, coined as good stress. However, acquired Piezo2 channelopathy fails to provide this ultrafast non-linear system protection against gravity under allostatic stress, leading to an energy generation switch due to OXPHOS depletion, resultant impaired metabolic regulation and impaired response to postural perturbations.

## 5. Conclusions

The quantum mechanical background in regard to non-synaptic neurotransmission within the central nervous system is emerging in the scientific literature [73] and its relevance is also emerging on the periphery [4,23]. However, the Piezo2-initiated novel ultrafast non-synaptic neurotransmission within the nervous system may also take the forms of a wormhole-like mechanism towards the hippocampus and motoneurons (Table 2). These wormhole-like phenomena may contribute to the ultrafast backbones of the brain axes. The absence or the impairment of these wormhole-like phenomena may result in a switch/miswiring in the nervous system and resultant impaired brain axes downstream. The theoretical proprioceptive wormhole-like mechanism in between the intrafusal Type Ia terminal Piezo2, and the hippocampus and motoneurons suggests that Piezo2 may serve as an anti-gravity fine/ultrafast modulator. Moreover, the microdamage of Piezo2 function may not only cause proton reversal (even electron reversal), but might be the equivalent of a Big Bang-like mechanism. The acquired Piezo2 channelopathy theory posits that there is no non-contact injury unless the functional microdamage of terminal somatosensory Piezo2 prevails, coined as the primary damage [10]. Acquired Piezo2 channelopathy may evolve not only in DOMS, but on Piezo2-containing somatosensory terminals contributing to proprioception under forced lengthening and allostatic stress, like in the skin, spine, and gut [10].

The link between the quantum gravity concept and quantum theory is short of being unequivocally proven. This scientific challenge seems not only a question for physicists, but the current author suggests that it is a challenge of medicine as well. Hence, the introduction of theoretical physics in mechanotransduction and proprioception seems to be a scientific demand in order to facilitate scientific development (Table 1).

Finally, the current author proposes that acute intensive exercise moments and DOMS provide the most attractive scientific opportunity to examine the system-wide effect of acquired Piezo2 channelopathy, since Piezo2-initiated pathways are microdamaged, degraded and degenerated by the time patients visit their doctors with their complaints and pain conditions.

One of the most fundamental structural questions is whether acquired Piezo2 channelopathy entails the direct microdamage of the plug-and-latch mechanism of Piezo2 [97] or the indirect dissociation of auxiliary subunit proteins of Piezo2 [10]. In the end it does not seem to matter, because both may result in a proton affinity switch or proton reversal when it should not happen.

Acquired Piezo2 channelopathy is an especially intriguing area of future research, because the resultant pathway switch within the nervous system, involving the hippocampus as the site for adult hippocampal neurogenesis, could be a key mechanism leading to accelerated aging and neurodegeneration.

## Figures and Tables

**Table 1:** Highlights.

Highlights
Principality of Piezo2 is theorized to come from proprioception-related mechanotransduction by fine or ultrafast regulation of force against gravity, transduced by protons (and electrons).
A theoretical wormhole-like mechanism may connect two distant points through a tunnel in spacetime, like the suggested axis between the intrafusal Piezo2-containing Type Ia proprioceptive terminal, and the hippocampus and motoneurons. These wormhole-like mechanisms are hypothesized to construct the underlying backbone of brain axes and be synchronized to the hippocampal theta rhythm under homeostatic state.
Piezo2 may not only be an (entropic) force sensor, but may fine-regulate force-from-proton as well.
Space, time and speed information is theorized to emerge from gravity in order to serve navigation and proprioception.
Evolutionarily conserved cryptochromes may not only contribute to photosynthesis and to the navigation of birds, but their photon absorption-induced radical pairs coupled to Piezo2-induced proton quantum tunneling propelled by OXPHOS are hypothesized to provide the negative energy for the theoretical wormhole-like mechanism in support of proprioception.
The impairment of this ultrafast non-synaptic long-distance synchronous neurotransmission is theorized to be reflected in acquired Piezo2 channelopathy and that is proposed to be analogous to a Big Bang-like mechanism.
Theoretical physics should have a place in science when it comes to mechanotransduction, or even more importantly when it comes to proprioception.

**Table 2:** Outstanding Questions.

Outstanding Questions
Is Piezo2 a force sensor? The current manuscript posits that Piezo2 may not only be an (entropic) force sensor, but might even fine-regulate force-from-proton in order to counteract gravity.
Does a (magnetic) wormhole-like mechanism exist in humans? The current manuscript suggests that this theorized ultrafast long-distance non-synaptic synchronous signaling should be contemplated although the quantum gravity concept is short of being unequivocally proven to be unified with quantum theory.
Is Piezo2 the principal mechanosensory ion channel responsible for proprioception? Mechanostransduction is hierarchical and at its top the current paper proposes that only Piezo2 is capable of initiating ultrafast long-distance proton-based quantum tunneling and even inducing wormhole-like mechanisms during ultradian events. These theoretical enigmatic features may explain Piezo2′s principality in proprioception. However, other ion channels certainly also contribute downstream, like ELKIN1 and auxiliary subunit proteins of Piezo2.
Is a Big Bang-like mechanism within humans? The current paper proposes that it is, in the acquired Piezo2 channelopathy and that this is the theorized principal gateway to pathophysiology or the one common root cause of aging initiation, coined as primary damage.
What is the critical structural site of Piezo2 where this functional impairment evolves as a Piezo2 channelopathy? Is it the plug-and-latch mechanism of Piezo2 that is microdamaged or do the auxiliary proteins of Piezo2 dissociate due to conformational changes? In the end it does not seem to matter, because both may result in a proton affinity switch or proton reversal when it should not happen.
Does theoretical physics have a place in science when it comes to mechanotransduction, or more importantly to proprioception? The current manuscript claims that it is time to invite theoretical physics to translate certain phenomena during mechanotransduction and proprioception, especially in reference to gravity, in order to facilitate scientific development.

## Data Availability

No new data were created or analyzed in this study.
